# miRNA-486-5p: signaling targets and role in non-malignant disease

**DOI:** 10.1007/s00018-022-04406-y

**Published:** 2022-06-22

**Authors:** Adrianna Douvris, Jose Viñas, Kevin D. Burns

**Affiliations:** 1 Division of Nephrology, Department of Medicine and Kidney Research Centre, The Ottawa Hospital Research Institute, University of Ottawa, 1967 Riverside Dr., Rm. 535, Ottawa, ON K1H 7W9 Canada; 2 Department of Cellular and Molecular Medicine, University of Ottawa, Ottawa, ON Canada

**Keywords:** microRNA, Extracellular vesicles, Apoptosis, Angiogenesis, Fibrosis, Ischemia reperfusion injury

## Abstract

MicroRNAs (miRNAs) are short non-coding RNAs, highly conserved between species, that are powerful regulators of gene expression. Aberrant expression of miRNAs alters biological processes and pathways linked to human disease. miR-486-5p is a muscle-enriched miRNA localized to the cytoplasm and nucleus, and is highly abundant in human plasma and enriched in small extracellular vesicles. Studies of malignant and non-malignant diseases, including kidney diseases, have found correlations with circulating miR-486-5p levels, supporting its role as a potential biomarker. Pre-clinical studies of non-malignant diseases have identified miR-486-5p targets that regulate major signaling pathways involved in cellular proliferation, migration, angiogenesis, and apoptosis. Validated miR-486-5p targets include *phosphatase and tensin homolog* (*PTEN*) and *FoXO1*, whose suppression activates phosphatidyl inositol-3-kinase (PI3K)/Akt signaling. Targeting of *Smad1/2/4* and *IGF-1* by miR-486-5p inhibits transforming growth factor (TGF)-β and insulin-like growth factor-1 (IGF-1) signaling, respectively. Other miR-486-5p targets include *matrix metalloproteinase-19* (*MMP-19*), *Sp5, histone acetyltransferase 1 (HAT1),* and *nuclear factor of activated T cells-5 (NFAT5)*. In this review, we examine the biogenesis, regulation, validated gene targets and biological effects of miR-486-5p in non-malignant diseases.

**Supplementary Information:**

The online version contains supplementary material available at 10.1007/s00018-022-04406-y.

## Introduction

MicroRNAs (miRNAs) are short non-coding RNAs, approximately 22–25 nucleotides long that were first identified in *Caenorhabditis elegans* in 1993 [1]. The human genome alone contains over 1500 miRNAs, although the functional significance of many remains uncertain [2]. miRNAs are highly conserved across species and potently regulate gene expression, as over 60% of human protein-coding genes are conserved targets of miRNAs [3]. The biogenesis of miRNAs is a multistep process that can be divided into canonical and non-canonical pathways, which have been reviewed elsewhere [4]. miRNAs function primarily by post-transcriptional gene silencing via the RNA-induced silencing complex (RISC) pathway in the cytoplasm, wherein the miRNA associates with an Argonaute protein in the RISC, and binds to the 3′ untranslated region (UTR) of its target mRNA [5]. Argonaute proteins associate with a GW182 protein that interacts with poly(A)-binding protein and a cytoplasmic deadenylase complex. Consequently, the miRNA–RISC complex mediates mRNA deadenylation and degradation, although inhibition of mRNA translation may also occur via an unclear mechanism [5]. miRNAs may also bind other regions of target genes, including the 5′UTR and coding sequences, and recent studies have identified a nuclear role as activators or silencers of gene transcription [6, 7]. miRNAs have garnered attention for their potential roles in disease diagnosis, pathogenesis, and therapy, as their expression is dysregulated in both malignant and non-malignant diseases [8–10].

miR-486-5p is a muscle-enriched miRNA [11] that circulates at relatively high levels in plasma [12], is enriched within small extracellular vesicles [13], and has been linked to signaling pathways involved in several cancers [9] as well as non-malignant conditions, such as skeletal muscle disorders [14, 15], ischemia–reperfusion injury (IRI) [16–18], and organ fibrosis [19–21]. Here, we review miR-486-5p biogenesis and regulation of expression, discuss circulating miR-486-5p as a potential biomarker in non-malignant diseases, and the role of exosomal transfer in experimental disease models. We also report major targets of miR-486-5p and affected biological pathways in non-malignant diseases, and highlight the controversies regarding downstream effects on cell and organ function. For information on the role of miR-486-5p in malignant diseases, the reader is referred to a recent comprehensive review [9].

## Biogenesis of miR-486-5p

miR-486-5p is highly conserved in mammals, and was first identified as a muscle-enriched miRNA whose expression is downregulated in Duchenne’s muscular dystrophy [22]. miR-486-5p is highly expressed in myoblasts during myogenesis, and in skeletal muscle [14], and is found at high levels in cardiac muscle, with moderate expression in lung, brain, liver, and bladder [11, 23, 24]. miR-486-5p is also a dominant erythroid miRNA, and its expression is upregulated in erythropoiesis [25, 26].

**Fig. 1:**
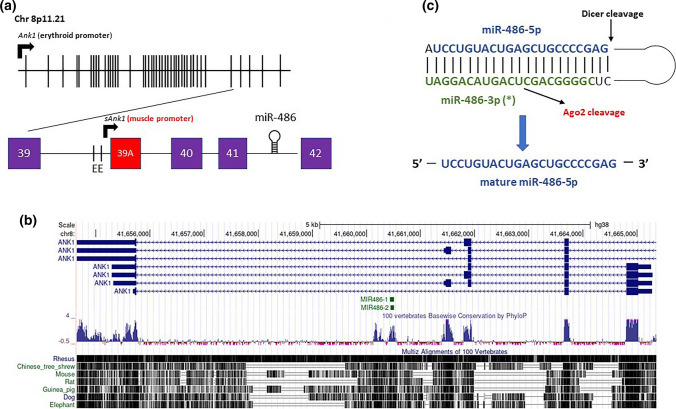
miR-486-5p biogenesis **a** miR-486-5p is transcribed from the last intron of the *Ank1* gene, located downstream from alternative exon (39A) of the muscle-specific *sAnk1* isoform **b** genomic organization of miR-486-5p and *Ank1* is conserved among mammals **c** pre-miR-486 requires Dicer cleavage, and also requires Argonaute-2 catalytic activity to remove the 3p star (*) strand, generating functional mature miR-486-5p (adapted from Refs. [11, 25])

miR-486 is first transcribed from an intron within the *ANK1* locus, located on chromosome 8p11.21 (Fig. 1). The *ANK1* locus encodes the Ankyrin 1 gene, which is primarily expressed in erythroid cells. The mouse genome contains miR-486a-5p and miR-486b-5p at this locus, both of which consist of the same mature sequence but are transcribed in opposite directions. Similarly, the human genome encodes miR-486-1 and miR-486-2, also consisting of the same mature sequence but transcribed in opposite directions. The biogenesis and expression of miR-486-5p discussed here refers to miR-486a-5p/miR-486-1, as these are both transcribed in the same direction as *Ank1.* Two miRNAs, miR-486-5p and miR-486-3p [9], are generated from opposite ends of the pre-miRNA hairpin [4]. miRNA strand selection is determined by intrinsic features of the miRNA duplex including the identity and thermodynamic stability of the 5′ nucleotides of each strand, but tissue-specific differences in strand selection (miRNA 5p/3p ratios) also support other regulatory mechanisms involving miRNA processing, duplex remodeling, and degradation [27]. miR-486 processing exhibits Argonaute-2-Slicer dependence, whereby Argonaute-2 catalytic activity is required to generate functional mature miRNA via slicing to remove the star (3p) strand; catalytically inactive Argonaute-2 results in miR-486-3p accumulation, miR-486 duplex arrest, and blunted miR-486-5p activity [25].

## miR-486-3p expression and function

The function and expression of the star strand, miR-486-3p, has been less well studied within different tissues. Within the hematopoietic system, miR-486-5p and miR-486-3p are highly expressed in erythroid cells [26]. However, their expression patterns differ in that miR-486-5p increases throughout the differentiation process, whereas miR-486-3p peaks earlier and then declines slightly [26]. Structurally, the pre-miR-486 miRNA/star duplex is perfectly paired and conserved across mammalian species, possibly reflecting conserved activity of miR-486-3p, although functional sensor assays used to validate miRNA activity showed that unlike mature miRNA-486-5p, miR-486-3p failed to repress its sensor [28]. Furthermore, immunoprecipitation of Argonaute proteins revealed that miR-486-3p associated with Argonautes 1 and 3, but functional sensor assays in wild type, Argonaute-2 knockout and Argonaute-2 catalytically inactive mouse embryonic fibroblasts showed that despite its accumulation, miR-486-3p did not exhibit gene regulatory activity [25].

Nonetheless, miR-486-3p target genes have been identified in malignant and non-malignant diseases [9], suggesting a role in gene regulation. In erythroid cells, miR-486-3p targets and downregulates the zinc finger protein *BCL11A*, associated with increased expression of the γ-globin gene and fetal hemoglobin synthesis [26]. miR-486-3p targets and downregulates the deacetylase *Sirtuin 2 (SIRT2),* resulting in decreased α-synuclein-induced toxicity in vitro, and suggesting that miR-486-3p may protect against Parkinson’s disease progression [29]. A recent systematic review of 34 studies found that miR-486-3p is among the most frequently altered miRNAs in patients with autism spectrum disorder (ASD) [30]. miR-486-3p was reported to be upregulated in the serum of patients with ASD, and targets and downregulates *AT-rich interaction domain 1B* (*ARID1B*) [31]*,* a gene mutated in ASD that also confers an ASD-like phenotype in *Arid1b* haploinsufficient mice [32]. Although the processing of pre-miR-486 and distribution of miR-486-5p and miR-486-3p within different tissues is not fully understood, these data support miR-486-3p as a functional miRNA with a potential role in human diseases. The remainder of this review will focus on miR-486-5p. For further information on miR-486-3p in human diseases, the reader is referred to a recent review [9].

## Regulation of miR-486-5p expression and cellular localization

Although Ank1 mRNA is primarily expressed in erythroid cells, an isoform (*sAnk1*/*Ank1.5*) containing a muscle-specific first exon and the last three exons of the *Ank1* erythroid gene is produced from an alternate promoter [33] (Fig. 1a, b). The tissue distribution of *sAnk1* mirrors that of miR-486-5p, enriched in heart and skeletal muscle [11]. Furthermore, there is increased expression of both *sAnk1* mRNA and miR-486-5p during myoblast differentiation [24]. In rat neonatal cardiomyocytes, Small et al.showed that myocardin-related transcription factor A (MRTF-A) induces the expression of both miR-486-5p and *sAnk1*. The *sAnk1* promoter also contains two conserved E boxes for MyoD transcriptional activity, and MyoD activity directly regulates *sAnk1* and miR-486-5p expression [24]. These data support the co-regulation of miR-486-5p and *sAnk1* and suggest that miR-486-5p is produced from the processing of *sAnk1* intronic RNA [11].

The regulation of miR-486-5p expression has derived largely from studies pertaining to malignancies, where its dysregulation is paradoxically associated with either tumor suppression or oncogenesis [9]. Epigenetic modifications of the *ANK1* promoter [34] and reduced p53 expression or activity [35] are associated with downregulation of miR-486-5p. The miR-486-5p promoter also contains a binding site for hypoxia-inducible-factor-1α (HIF-1α). HIF-1α overexpression activates the miR-486-5p promoter in HeLa cells, and induces expression of miR-486-5p in prostate cancer cell lines, suggesting that hypoxia stimulates its transcription [36]. The effect of hypoxia on miR-486-5p expression is not limited to malignant cells as hypoxic rat cardiomyocytes also exhibit upregulated miR-486-5p [37]. miR-486-5p expression is also regulated at the post-transcriptional level. For example, long non-coding RNAs (lncRNAs) can directly bind to miR-486-5p, inhibiting its expression and activity in experimental models [21, 38, 39].

Traditionally, miRNA biogenesis is a multi-step process involving nuclear and cytosolic components, with the classical function of mature miRNA being post-transcriptional gene silencing in the cytoplasm via miRISC and the 3′ UTR of the target gene [7]. Studies with high-throughput profiling techniques have identified mature miRNAs that are enriched in cell nuclei [40–42], and RISC components including Argonaute-2 and trinucleotide repeat-containing gene 6A protein (TNRC6) have been identified in the nucleus and thus likely play a role in miRNA nuclear transport and activity [43, 44]. Nuclear miRNAs can (1) mediate post-transcriptional gene silencing to downregulate the expression of long non-coding RNAs, (2) interact with pri-miRNAs to inhibit their maturation; (3) activate or suppress gene transcription by interacting with gene promoters in association with Argonaute-2; and (4) induce gene expression by enhancer activation, which may also require Argonaute-2 [7].

The cellular localization of mature miR-486-5p is indeed not limited to the cytoplasm. Deep sequencing of the nuclear and cytoplasmic pools of small RNAs from a human nasopharyngeal carcinoma cell line identified 339 nuclear and 324 cytoplasmic miRNAs, the majority of which overlap, including miR-486-5p [42]. The large degree of overlap suggests that miRNAs are imported into the nucleus. Viñas et al. administered intravenous lipid-encapsulated miR-486-5p mimic to mice with bilateral kidney IRI and by *in-situ* hybridization revealed that miR-486-5p localized to both cytoplasm and nuclei of cortical tubular cells [45]. Thus, it is conceivable that miR-486-5p may exert biological effects in the nucleus via one or more of the identified nuclear roles of miRNAs [7], such as post-transcriptional gene silencing of other non-coding RNAs or regulation of protein-coding gene expression through direct interaction with promoter or enhancer sites.

## Circulating miR-486-5p and extracellular vesicles

Circulating miRNAs are stable in plasma and resistant to nuclease digestion [46]. An estimated 90% of circulating miRNAs are present in a non-membrane bound form, associated with the Argonaute-2 ribonucleoprotein complex, whereas a smaller proportion are found within small extracellular vesicles, such as exosomes (50–150 nm diameter) [46, 47]. miR-486-5p is abundant in human plasma [12, 48], where it circulates freely or within exosomes [49]. However, as an erythropoietic miRNA, miR-486-5p is also released by red blood cell hemolysis, thereby increasing miR-486-5p levels in hemolyzed samples. This feature can thereby complicate the interpretation of circulating miR-486-5p levels in biomarker studies [50, 51].

miR-486-5p is differentially expressed in human plasma or serum in a wide range of conditions including, but not limited to solid tumor malignancies [9], sepsis [52], primary muscle diseases (e.g., Duchenne muscular dystrophy [22]), cardiorespiratory diseases (e.g., chronic heart failure [19], cystic fibrosis [53]), diabetic kidney disease [54], osteoarthritis [55], neurological conditions (e.g., vascular dementia [56], Huntington’s disease [57], ASD [58]), and various endocrine disorders (e.g., metabolic syndrome [59], childhood obesity [60], type 2 diabetes mellitus [61], polycystic ovary syndrome [62], and recurrent miscarriage [63]). Accordingly, circulating levels may have utility for diagnosis or prognosis in a variety of diseases (outlined in Supplementary Table 1). Although these studies provide data on clinical associations, further research is required to determine the diagnostic and prognostic potential of circulating miR-486-5p. Of the studies outlined in Supplementary Table 1, the diagnostic accuracy of circulating miR-486-5p was evaluated only in type 2 diabetes [54], vascular dementia [56], and outcomes of embryo transfer with in vitro fertilization (IVF) [63]. Regmi et al.evaluated serum miRNAs in patients with diabetic kidney disease, revealing that miR-486-5p was downregulated, with a receiver operating characteristic area under the curve (ROC-AUC) of 0.853 [54]. In patients with vascular dementia due to cerebral small vessel disease, plasma miR-486-5p had a sensitivity of 75%, specificity of 83%, and ROC–AUC of 90% as a diagnostic marker [56]. In a study of embryo transfer outcomes in IVF, a plasma miRNA signature that included miR-486-5p had a sensitivity of 100% and specificity of 83% for recurrent miscarriage, but sensitivity of only 68.1% and specificity of 54% for successful outcome [63]. Given the variety of non-malignant diseases with dysregulated circulating miR-486-5p levels, its potential as a non-invasive diagnostic and prognostic biomarker warrants further study.

miR-486-5p is enriched within exosomes, which are important mediators of intercellular communication [64] and have shown protective effects in experimental models of organ injury [16, 17, 65, 66]. Indeed, miR-486-5p is among the most abundant miRNAs in both human adipose and bone marrow mesenchymal stem cell (BMSC)-derived exosomes, and over-represented compared to its expression within the parent cells [13]. Furthermore, human cord blood endothelial colony-forming cell (ECFC)-derived exosomes are enriched in miR-486-5p, with levels that are markedly higher compared to ECFC-derived microparticles (100–1000 nm diameter) [16]. Mechanisms involved in selective cargo loading of miRNA and other RNA species into extracellular vesicles remain unclear. However, factors that influence the miRNA profile within extracellular vesicles include the pathophysiological state of the source cell, RNA properties (such as small size, affinity for membrane lipids, and cytoplasmic localization), RNA sequence motifs, post-transcriptional modifications, and associations with RNA binding proteins [64].

## Targets of miR-486-5p

Based on 3′UTR sequence homology (http://mirdb.org/cgi-bin/search.cgi), more than 300 predicted miR-486-5p targets have been identified. However, not all predicted targets have been validated by demonstrating a direct interaction between miR-486-5p and the gene transcript 3′ UTR, typically done by luciferase reporter assay. Below, we review key validated targets of miR-486-5p in non-malignant diseases.

## Skeletal muscle *PTEN* and *FoXO1*

*Phosphatase and tensin homolog (PTEN)* is a tumor suppressor protein whose expression is tightly regulated at multiple levels [67], and represents the original validated target of miR-486-5p [11]. As a lipid phosphatase, *PTEN* negatively regulates the phosphatidyl-inositol-3-kinase (PI3K)/Protein kinase B (Akt) signaling pathway by de-dephosphorylating the intracellular messenger and PI3K product phosphatidyl-inositol-triphosphate (PIP3) to PIP2 [68]. Consequently, *PTEN* inhibits several cellular functions including proliferation, migration, survival, and angiogenesis [68, 69].

*FoxO1* belongs to the family of forkhead transcription factors (FoxOs), which are involved in insulin and insulin-like growth factor-1 (IGF-1) signaling, thus affecting cell growth, proliferation, differentiation, apoptosis, oxidative stress, ageing, and metabolism [70]. *FoxO1* is a negative regulator of Akt signaling, and its activity is also inhibited via Akt-mediated phosphorylation [71].

Small et al. [11] first identified *PTEN* and *FoxO1* as miR-486-5p targets, by luciferase reporter assay. In mice, an inverse correlation was shown between the expression of miR-486-5p and *PTEN* in postnatal cardiac growth. Furthermore, miR-486-5p overexpression in cardiomyocytes downregulated endogenous *PTEN* and *Fox01* protein expression and increased p-Akt (at Ser473). These data implicate miR-486-5p as a promoter of cardiac muscle growth by targeting and downregulating *PTEN* and *Fox01*, thereby activating PI3K/Akt signaling [11].

**Fig. 2:**
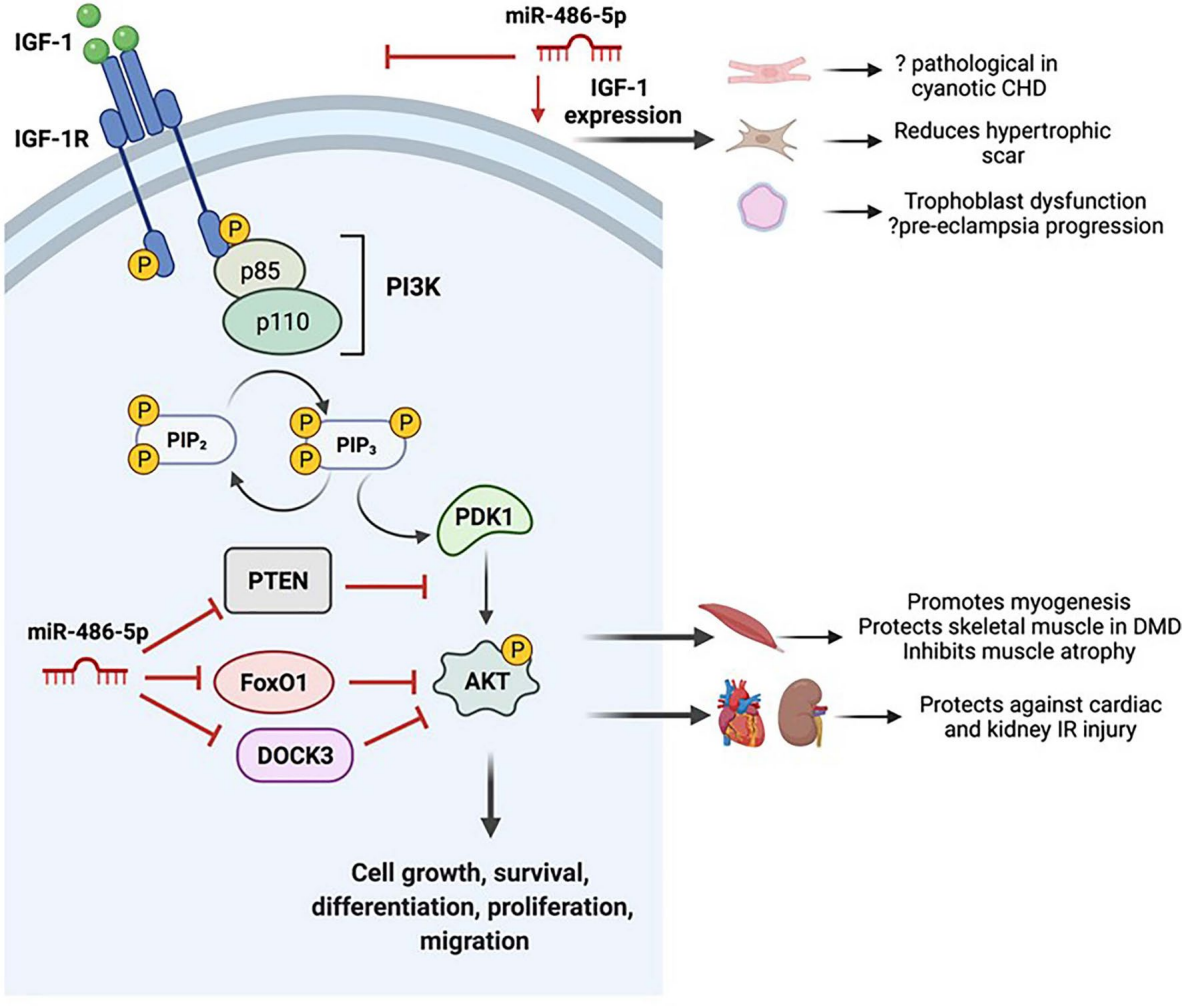
miR-486-5p can activate or suppress PI3K/Akt signaling via its targets. miR-486-5p targets *PTEN, DOCK3,* and *FoxO1* to activate PI3K/Akt signaling, and confers protective effects in skeletal muscle disorders, and kidney and cardiac IR injury (top). miR-486-5p targets *IGF-1* to inhibit PI3K/Akt signaling, with possible negative consequences in cyanotic congenital heart disease and pre-eclampsia. *CHD* congenital heart disease, *DMD* Duchenne muscular dystrophy, *IGF-1* insulin-like growth factor 1, *IR* ischemia reperfusion, *PI3K* phosphatidylinositol-3-kinase, *PTEN* phosphatase and tensin homolog (created with BioRender.com)

*PTEN* and PI3K/Akt signaling has also been implicated in primary muscle disorders, including Duchenne muscular dystrophy (DMD), caused by mutations in the *dystrophin* gene [72]. miR-486-5p expression is downregulated in skeletal muscle of patients with DMD compared to healthy controls [22] and in dystrophin-deficient muscles in mice [23]. Dystrophin-deficient animals have increased *PTEN* expression and decreased Akt phosphorylation within dystrophin-deficient muscle tissue [73], and modulation of Akt signaling improves muscle function in these models [74]. At the cellular level, miR-486-5p inhibition is detrimental to myoblasts, resulting in reduced myotube formation, failed migration, increased caspase-3/7 levels and enhanced apoptosis [14]. In human myotubes that overexpress *DOCK3* (a validated target of miR-486-5p in skeletal muscle), *PTEN* expression is increased, associated with reduced Akt phosphorylation, higher expression of activated caspases-3/7 and increased myotube apoptosis [23]. These results provide strong evidence that miR-486-5p modulates these pathways via targeting *PTEN* and *DOCK3* (Fig. 2).

The dynamic expression of miR-486-5p in normal muscle regeneration is critical for its regenerative effect [14]. A comparison of wild type and *dystrophin*-deficient mice subjected to cardiotoxin-induced tibialis anterior muscle injury revealed that miR-486-5p expression is transiently upregulated after injury, with normal muscle regeneration, while *dystrophin*-deficient mice have reduced miR-486-5p expression following injury and impaired muscle regeneration. The authors also evaluated muscle regeneration in transgenic mice that overexpress miR-486-5p exclusively in heart and skeletal muscle. Transgenic mice with muscle-specific miR-486-5p overexpression are viable, with no significant phenotypic differences from wild-type controls at 6 months of age, other than slight weight gain. However, when subjected to cardiotoxin-induced skeletal muscle injury, the transgenic mice displayed slowed muscle regeneration and altered architecture, with increased multinucleated myofibers and a higher proliferative index [14]. Post-skeletal muscle injury, mice with muscle-specific miR-486-5p overexpression had decreased expression of target genes *PTEN* and *FoxO1* (compared to wild type mice) along with downregulation of cyclin-dependent kinase inhibitors p21and p27 [14], which are *FoxO1* targets [75]. These findings suggest that maintaining high miR-486-5p expression (rather than dynamic miR-486-5p expression) influences muscle satellite cell kinetics and fusion associated with targeting of *PTEN* and *FoxO1*, resulting in delayed and abnormal skeletal muscle regeneration [14].

In addition to myogenic differentiation and DMD, miR-486-5p protects against muscle wasting through targets *PTEN* and *FoxO1* [15, 66]. In experimental chronic kidney disease (CKD), the catabolic environment increases glucocorticoid production and suppresses insulin signaling, resulting in decreased PI3K/Akt activity [76, 77]. Suppression of Akt signaling induces *FoxO1* dephosphorylation, which translocates to the nucleus and activates expression of E3 ubiquitin ligases responsible for muscle proteolysis [77]. In mice with muscle-specific gene deletion of *FoXO1*, Xu et al. reported protection against CKD-induced muscle wasting [15]. In vivo, muscle miR-486-5p levels were decreased in CKD mice, and electroporation of miR-486-5p mimic into muscle improved mass, and decreased expression of ubiquitin E3 ligases, *FoxO1* and *PTEN* [15]. In dexamethasone-treated cultured myotubes, Li et al.demonstrated that BMSC-derived exosomes (enriched in miR-486-5p) improved myotube quantity, reduced expression of muscle atrophy markers, and downregulated the nuclear translocation of *FoxO1*, whereas these effects were blunted using exosomes treated with miR-486-5p inhibitor [66]. Accordingly, these data suggest that miR-486-5p is a potential therapeutic strategy for disorders of muscle catabolism via its targeting of *PTEN* and *FoxO1*.

## Targeting of *PTEN* and PI3K/Akt signaling in cardiac ischemia

Acute myocardial infarction is associated with IRI [78] and coronary microembolization [79], which lead to myocardial cell apoptosis. In a swine model of coronary microembolization, activation of the *PTEN*/Akt pathway contributes to cardiomyocyte apoptosis [80]. In rats with coronary microembolization, ventricular miR-486-5p expression is downregulated, and its overexpression activates PI3K/Akt signaling by reducing *PTEN* expression, resulting in decreased cardiomyocyte apoptosis, reduced microinfarct area and improved cardiac function [81]. In a preclinical study of myocardial IRI, hypoxia/re-oxygenation triggers cardiomyocyte apoptosis with increased *PTEN* expression and reduced miR-486-5p expression [17]. Exosomes derived from rat BMSCs overexpressing miR-486-5p have a pronounced inhibitory effect on *PTEN* expression, leading to Akt activation, apoptosis reduction and significant reduction in myocardial infarct size compared to unaltered exosomes [17].

Bei et al. demonstrated that endogenous miR-486-5p was downregulated in cardiac tissue from mice subjected to IRI [18]. Injection of adeno-associated virus 9 (AAV9)-expressing miR-486-5p to mice subjected to cardiac IRI significantly reduced infarct size and cardiomyocyte apoptosis at 24 h, preserved cardiac function and reduced cardiac fibrosis at 3 weeks. Long term cardiac remodeling and dysfunction (at 6 weeks) was also prevented [18]. At 3 weeks after IRI, there was decreased expression of *PTEN* and *FoxO1* in cardiac tissue of mice treated with miR-486-5p. Moreover, cardiomyocyte-specific expression of miR-486-5p suppressed apoptosis, attenuated cardiac dysfunction and fibrosis at 3 weeks, and downregulated *PTEN* and *FoxO1* expression. On the other hand, miR-486-5p inhibition by miR-486-5p sponge AAV9 in cardiac IRI did not further increase infarct size at 24 h or worsen cardiac dysfunction at 3 weeks [18]. Of interest, swimming exercise upregulated miR-486-5p expression in isolated cardiomyocytes from mouse heart tissue, downregulated cardiac *PTEN* and *FoxO1* expression, and protected against cardiac IRI [18]. Furthermore, miR-486-5p knock out mice subjected to exercise prior to cardiac IRI had significantly increased infarct size at 24 h compared to wild type controls [18]. miR-486-5p mimic suppressed apoptosis in cultured rat neonatal and human induced pluripotent stem cell-derived (hiPSC) cardiomyocytes, along with decreased expression of *PTEN* and *FoxO1* and activation of Akt signaling. Finally, while anti-miR-486-5p increased apoptosis in rat cardiomyocytes, silencing either *PTEN* or *FoxO1* in anti-miR-486-5p-transfected cardiomyocytes attenuated the pro-apoptotic effect of miR-486-5p inhibition [18].

These compelling results suggest that exosomal transfer or direct administration of miR-486-5p protects against myocardial IRI by targeting *PTEN* and *FoxO1*, thereby activating Akt signaling and suppressing cardiomyocyte apoptosis (Fig. 2). Furthermore, these data also support an important role of miR-486-5p for the cardioprotective benefit of exercise in cardiac IRI.

## *Matrix metalloproteinase-19 (MMP-19)* and promotion of angiogenesis in myocardial infarction

Besides targeting *PTEN*/PI3K/Akt signaling, miR-486-5p may protect against myocardial ischemic injury via other pathways. De novo angiogenesis may protect injured cardiomyocytes, prevent adverse cardiac remodeling and thereby reduce the risk of heart failure after myocardial infarction [82]. In BM-MSCs under hypoxia, the expression of miR-486-5p and the pro-angiogenic vascular endothelial growth factor (VEGF) is upregulated [83]. miR-486-5p overexpression increases VEGF mRNA, and miR-486-5p inhibition downregulates VEGF mRNA and secretion, suggesting that miR-486-5p confers a proangiogenic effect [83].

Matrix metalloproteinase 19 (*Mmp-19*) is an inhibitor of angiogenesis whose expression is downregulated in invasive carcinomas [84]. *Mmp-19*-deficient mice display earlier onset tumor angiogenesis [85] and *Mmp-19* reduces endothelial cell angiogenesis by proteolytic cleavage of plasminogen, generating angiostatin-like fragments as endogenous angiogenesis inhibitors [86]. Li et al.studied the effect of exosomal miR-486-5p on angiogenesis after myocardial infarction in mice and non-human primates [65]. In mice with myocardial infarction, intra-myocardial administration of hypoxia-preconditioned MSC-derived exosomes, enriched in miR-486-5p, reduced infarct size, improved left ventricular ejection fraction and increased vascular density after 4 weeks. Exosomes also increased vessel sprouting from isolated aortic rings, further supporting their pro-angiogenic effect. Hypoxia-preconditioned exosomes from MSCs treated with anti-miR-486-5p failed to promote myocardial repair and angiogenesis, suggesting that their protective effect is mediated in part by miR-486-5p. RNA sequencing of mouse myocardial tissues identified *Mmp-19* as significantly downregulated in the exosome-treated hearts. *Mmp-19* is highly expressed in cardiac fibroblasts, and is a direct target of miR-486-5p by luciferase reporter assay. Furthermore, pathway analysis revealed that VEGF signaling is upregulated in myocardial tissue from exosome-treated mice, and levels of uncleaved VEGF-A are higher in fibroblasts overexpressing miR-486-5p or with silencing of *Mmp-19* [65], thus linking miR-486-5p to angiogenesis through its target *Mmp-19*.

Further studies in a non-human primate model of myocardial infarction revealed that hypoxia-preconditioned exosomes (enriched in miR-486-5p) promote cardiac angiogenesis, reduce infarct size, and improve cardiac function [65]. After 17 month follow-up, increased vascular and arterial density was found within exosome-treated hearts, indicating long-lasting cardiac angiogenesis. Intramyocardial injection of miR-486-5p-overexpressing exosomes improves cardiac function, reduces infarct size, and increases vascular density [65]. These results suggest that miR-486-5p promotes angiogenesis and functional myocardial recovery in two pre-clinical models of myocardial infarction. The pro-angiogenic effect may occur via a paracrine mechanism from fibroblasts that targets *Mmp-19* expression, resulting in decreased cleavage of extracellular VEGF-A.

## Targeting of the PTEN/Akt pathway in kidney ischemic injury

 Acute kidney injury (AKI) refers to a rapid decline in kidney function and is a common complication of hospitalization, affecting up to 20% of patients with a higher prevalence in critical care settings [87]. In-hospital mortality rises with increasing severity of AKI with a rate for severe AKI up to 50%[88]. Patients who recover from AKI are at risk of adverse outcomes including new or progressive CKD, and kidney failure [89]. Yet, no effective treatments for AKI exist and preventative measures are limited [90].

The role of miR-486-5p has been evaluated in the context of kidney IRI, an experimental model of human ischemic AKI characterized by tubular cell damage and apoptosis/necrosis, as well as endothelial cell dysfunction and loss [91]. Human cord blood ECFC-derived exosomes, highly enriched in miR-486-5p, suppress apoptosis of cultured human endothelial cells subjected to hypoxia/reoxygenation by transfer of miR-486-5p, targeting *PTEN* and activating Akt signaling [16, 92]. In mice with kidney IRI, ECFC-derived exosomes decrease kidney *PTEN* expression (and activate Akt), associated with reduced apoptosis, histologic injury and improved kidney function [16]. These data suggest that ECFC-derived exosomes protect against kidney IR injury via transfer of miR-486-5p.

A recent study by Viñas et al. examined the effects of direct intravenous administration of lipid-encapsulated miR-486-5p mimic to mice with ischemic AKI, and evaluated the transcriptome of kidney proximal tubules and endothelial cells [45]. miR-486-5p mimic significantly improved kidney function and decreased histological injury and apoptosis in mice subjected to kidney IRI, and prevented the proximal tubular activation of genes commonly associated with ischemic injury. Kidney *PTEN* protein expression was decreased by miR-486-5p, associated with activation of Akt.

Importantly, the protective effects of miR-486-5p in ischemic AKI may involve other distinct miR-486-5p targets besides *PTEN*. In particular, distinct proximal tubular genes associated with apoptosis and the tumor necrosis family (TNF) pathway are significantly downregulated by miR-486-5p 24 h after IRI [45]. Furthermore, RNA sequencing of kidney proximal tubular cells and endothelial cells revealed only few known miR-486-5p targets downregulated by mimic 24 h after IRI. Notably, *in-situ* hybridization revealed that miR-486-5p localizes to the cytoplasm and nucleus of kidney cortical tubular cells [45], raising the possibility that miR-486-5p may directly regulate gene transcription at the nuclear level, in addition to its effects via 3′UTR targeting.

## Targeting of *NFAT5* in chronic kidney disease: diabetic nephropathy

CKD is defined by the presence of abnormalities of kidney structure or function for at least 3 months [93]. Diabetes mellitus is the leading cause of CKD world-wide, and diabetic nephropathy (DN) affects approximately 30% of patients with diabetes [94]. The pathophysiology of DN involves hemodynamic and metabolic factors including increased intraglomerular pressure and hyperfiltration, and the production of advanced glycation end-products. This promotes the generation of growth factors and hormones, such as transforming growth factor (TGF)-β and angiotensin II, reactive oxygen species, and inflammatory mediators, all of which contribute to kidney histological changes including glomerular basement membrane thickening, extracellular matrix (ECM) deposition within the mesangium, proliferative changes, and ultimately, tubulointerstitial fibrosis and glomerulosclerosis [94, 95].

The *nuclear factor of activated T-cells* (*NFAT*), the substrate for calcineurin, represents a family of calcium-dependent transcription factors. *NFAT5* is ubiquitously expressed in all tissue types, and is an important gene regulator in organs with high hyperosmotic pressure risk, such as kidneys, heart, and brain [96]. However, *NFAT5* can have a pathogenic role in disease as it regulates the expression of pro-inflammatory cytokines [96], increases nuclear factor (NF)-κB activity [97], and increases the expression of genes involved in vascular smooth muscle cell and macrophage migration, platelet activation, and angiogenesis [96]. In experimental DN, the *NFAT* family of transcription factors is required for ECM protein accumulation and glomerular hypertrophy [98], and *NFAT* inhibition reduces podocyte injury [99] and renal fibrosis [100].

In this context, Duan et al. demonstrated that *NFAT5* is a validated target of miR-486-5p in DN [21]. In addition, miR-486-5p is a downstream target of lnc-ISG20 RNA: lnc-ISG20 expression increases in kidneys of diabetic mice and in mesangial cells cultured in high glucose, while miR-486-5p decreases; *NFAT5* expression also increases in both models [21]. *NFAT5*-induced Akt phosphorylation promotes fibrosis by increasing the expression of collagen, fibronectin and TGF-β in mesangial cells cultured in high glucose. In mice with DN, lnc-ISG20 overexpression promotes kidney fibrosis via Akt phosphorylation, while *NFAT5* knockdown prevents fibrosis [21]. Thus, lnc-ISG20 downregulates miR-486-5p expression, resulting in increased *NFAT5* expression, Akt activation, and increased expression of pro-fibrotic genes [21]. These data support an anti-fibrotic role of miR-486-5p in diabetic nephropathy.

Of note, the effect of glucose on miR-486-5p expression may be cell-specific. Although high glucose downregulates miR-486-5p expression in mesangial cells [21], exposure of human adipose tissue-derived MSCs to high glucose upregulates miR-486-5p expression, which inhibits cellular proliferation by targeting and downregulating *SIRT1* [101], a deacetylase that regulates gene expression and is implicated in insulin sensitivity in type 2 diabetes [102]. *SIRT1* expression is downregulated in adipose tissue of patients with diabetes [102], and in endothelial progenitor cells exposed to high glucose [103]. Bouchareychas et al. showed that miR-486-5p is upregulated in monocytes from hyperglycemic mice, in exosomes derived from BM-derived macrophages exposed to hyperglycemia, and in plasma-derived exosomes from patients with diabetes and peripheral arterial disease [104]. Thus, although miR-486-5p may protect against kidney fibrosis by targeting *NFAT5* in DN, its upregulation in other cell types in the context of hyperglycemia may have adverse consequences.

## Targeting of *IGF-1*

*Insulin-like growth factor-1 (IGF-1)* is a polypeptide trophic factor whose biological actions are mediated by the IGF-1 receptor, a tyrosine kinase that phosphorylates intracellular proteins to activate multiple signaling pathways, including PI3K/Akt and mitogen-activated protein kinase (MAPK) [105]. Consequently, *IGF-1* is involved in several cellular functions including survival, growth, and differentiation. Targeting of *IGF-1* by miR-486-5p in malignant diseases has been reported to reduce cancer cell growth and migration, and stimulate apoptosis [35, 106].

Studies of miR-486-5p and *IGF-1* signaling in non-malignant diseases have been limited to in vitro models, and reveal conflicting effects on proliferation, migration, and apoptosis. *IGF-1* is an essential regulator of cardiac structure and homeostasis [107] and reduced serum levels of *IGF-1* are found in patients with cyanotic congenital heart disease, who experience chronic hypoxemia [108]. Erythrocyte levels of miR-486-5p levels are increased in pediatric patients with cyanotic congenital heart disease compared to healthy controls [109]. Fan et al. showed that miR-486-5p expression is upregulated in rat cardiomyocytes exposed to hypoxia, and demonstrated *IGF-1* is a direct target of miR-486-5p by luciferase reporter assay. Downregulation of miR-486-5p increases *IGF-1* expression and cell viability, and suppresses hypoxia-induced cardiomyocyte apoptosis, whereas silencing of *IGF-1* has the opposite effect [37]. The data suggest that miR-486-5p may have a pathological role in cyanotic congenital heart disease by targeting *IGF-1*, thereby promoting cardiomyocyte apoptosis (Fig. 2).

The role of miR-486-5p and *IGF-1* targeting has also been studied in pre-eclampsia, a serious pregnancy-specific complication mediated by placental dysfunction. Human placental microvascular endothelial cell-derived exosomes are enriched in miR-486-5p [110] and levels are further upregulated following exposure to hypoxia/reoxygenation. Administration of hypoxia-exposed exosomes to trophoblast cells results in transfer of miR-486-5p, associated with decreased cell viability, proliferation, migration and invasion, via targeting of *IGF-1* [110]. These data suggest that miR-486-5p may have a pathological role in the development and progression of pre-eclampsia by causing trophoblast dysfunction*.* Similarly, an in vitro study in hypertrophic scar fibroblasts showed that miR-486-5p overexpression inhibits cell viability, migration and expression of collagens and promotes apoptosis by targeting *IGF-1* [111]. Interestingly, miR-486-5p has opposing biological effects in this model, suppressing PI3K/Akt signaling through *IGF-1*, in contrast to skeletal muscle [15, 23] and models of cardiac IRI [17, 81] Thus, cell-specific factors influence the regulation of miR-486-5p expression and its affected target genes, which ultimately determine biological effects. Indeed, in malignant diseases miR-486-5p has been reported to have both oncogenic and tumor suppressor roles [9].

## Targeting ***SMAD1/2/4*** and inhibition of TGF-β signaling

TGF-β belongs to a superfamily of related growth factors and comprises three isoforms in mammals (TGF-β1, TGF-β2, TGF-β3), all of which bind the TGF-β receptor 2 (TGFR2) to recruit TGFR1 and activate signaling [112]. TGF-β signaling is the primary driver of tissue fibrosis, but also has effects on cell proliferation, differentiation, apoptosis, and immunity [112]. TGF-β signaling activates *Smad*-based pathways by phosphorylation and activation of *Smad2/3* by the TGFR1, resulting in nuclear translocation of the *Smad* complex and transcription of pro-fibrotic genes, such as smooth muscle actin (α-SMA), collagens, and fibronectin [112]. TGF-β also interacts with non-*Smad*-based signaling including the MAPK and PTEN/PI3K/Akt pathways. Ultimately, TGF-β signaling through *Smad* and non-*Smad*-based pathways induces fibrosis via myofibroblast activation, excessive ECM production, and inhibition of ECM degradation [112].

In mice with pulmonary fibrosis, lung miR-486-5p levels diminish, and decreased levels are found in the serum and lung tissue of patients with silicosis and idiopathic pulmonary fibrosis [20]. Administration of miR-486-5p attenuates pulmonary fibrosis in mice exposed to silica or bleomycin [20]. In cultured mouse fibroblasts miR-486-5p directly targets *Smad2*, inhibits TFG-β-induced expression of pro-fibrotic genes and reduces fibroblast proliferation [20]. In human hypertrophic scar fibroblasts, miR-486-5p targets *Smad2* to inhibit proliferation and induce apoptosis [113]. These data support an anti-fibrotic role for miR-486-5p in models of pulmonary fibrosis and hypertrophic scar.

Epithelial–mesenchymal transition (EMT) is another TGF-β-mediated mechanism that contributes to fibrosis [112], and is implicated in development of posterior capsular opacification, (also known as secondary cataract), a complication of cataract surgery. In vitro studies using cultured human lens epithelial cells reported that miR-486-5p expression is downregulated in TGF-β2-induced lens cells, and overexpression of miR-486-5p reduces proliferation, migration, and EMT by targeting *Smad2* and *Smad4*, suggesting that miR-486-5p may prevent the progression of cataracts [114, 115].

Heart failure, a common end-stage manifestation of cardiac diseases, is associated with cardiac remodeling, characterized by cardiomyocyte hypertrophy, apoptosis, cardiac fibroblast activation and ECM deposition [116]. Serum IgE levels are elevated in patients with heart failure, and the IgE receptor (FCεR1) is upregulated in heart tissue from these patients [117]. Zhao et al. showed that blocking the IgE-FCεR1 pathway alleviates pathological cardiac remodeling in two distinct mouse models of heart failure [117]. Treatment of rat cardiac fibroblasts with IgE results in fibroblast activation, matrix protein production, and upregulation of TGF-β signaling in an FCεR1-dependent manner [117].

Zhao et al. [19] subsequently reported that miR-486-5p is downregulated (and its predicted target *Smad1* is upregulated) in mouse cardiac fibroblasts treated with IgE in an FCεR1-dependent manner, while the expression of collagens and α-SMA is also increased. Luciferase reporter assay revealed that *Smad1* is a direct target of miR-486-5p, while miR-486-5p mimic decreases expression of *Smad1*/p-*Smad1* in primary mouse cardiac fibroblasts. *Smad1* also promotes activation and collagen expression in cardiac fibroblasts treated with IgE. These data support a role for the miR-486-5p/*Smad1* pathway in IgE-induced collagen expression, and suggest that miR-486-5p protects against cardiac fibrosis by targeting and downregulating *Smad1*.

**Fig. 3:**
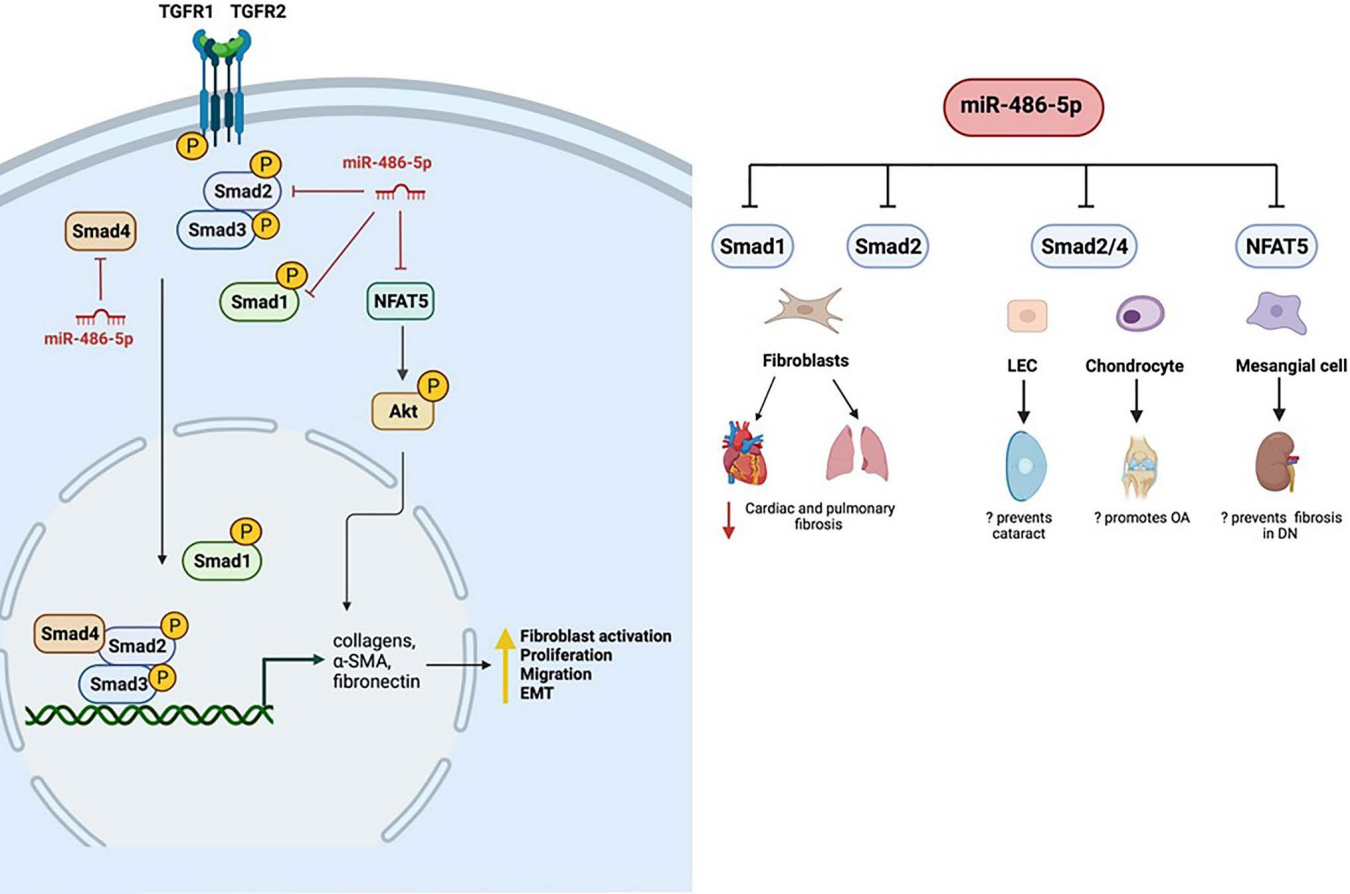
miR-486-5p targets *Smad-*dependent TGF-β signaling. miR-486-5p confers protective effects in models of cardiac and pulmonary fibrosis, hypertrophic scar, and cataract progression but may have a role in osteoarthritis pathogenesis. *EMT* epithelial-to-mesenchymal transition, *NFAT-5* nuclear factor of activated T cells-5, *OA* osteoarthritis, *α-SMA* α-smooth muscle actin, *TGF-β* transforming growth factor β (created with BioRender.com)

Although targeting of TGF-β/*Smad* signaling by miR-486-5p may protect against select fibrotic diseases, other processes may be negatively impacted. Shi et al. reported that patients with osteoarthritis (the most common form of arthritis caused by degeneration of joint cartilage) exhibit low levels of *Smad2* and elevated miR-486-5p levels in cartilage tissue compared to control patients. In cultured human chondrocytes, miR-486-5p targets *Smad2* and decreases cell proliferation and type II collagen expression. These data suggest that miR-486-5p may promote osteoarthritis progression by targeting *Smad2* [118] (Fig. 3). However, using collagen-induced arthritic mice as a model for the inflammatory autoimmune disease rheumatoid arthritis, Chen et al.demonstrated that exosomes containing miR-486-5p alleviate disease severity by decreasing the expression of *Tob1*, an antiproliferative protein that interacts with *Smad* family proteins [119], inducing osteoblast differentiation [120]. Thus the role of miR-486-5p in arthritis is complex and may differ based on arthritis etiology.

## Targeting *Sp5*/Wnt signaling

Successful wound healing requires cellular proliferation, migration, and angiogenesis [121]. The Wnt/β-catenin pathway involves evolutionary conserved signaling that is essential for cell fate and organization during embryogenesis, and plays a role in adult tissue homeostasis and regeneration [122]. The *Sp5* transcription factor is a Wnt target gene that negatively regulates Wnt signaling by transcriptional repression of downstream genes, such as *p21* and cyclin-D2, both of which regulate cellular proliferation, and the latter has also been implicated in endothelial cell repair [123].

Lu et al.investigated the role of adipose-derived stem cell-secreted extracellular vesicles, enriched in miR-486-5p, on cutaneous wound healing and the validated target *Sp5* [121]. In vitro experiments revealed that vesicle transfer of miR-486-5p enhances proliferation and migration of human skin fibroblasts, and stimulates proliferation, migration, and angiogenesis of human microvascular endothelial cells by inhibiting the expression of *Sp5*, thereby increasing cyclin-D2 expression. Thus, the relationship between miR-486-5p targeting of the transcriptional repressor *Sp5* and endothelial repair warrants further exploration in other models of organ injury and recovery that require de novo angiogenesis.

## miR-486-5p targets *Histone acetyltransferase 1*

Not all pre-clinical data demonstrate protective effects of miR-486-5p in disease. In this regard, histone acetylation is an epigenetic modification that changes chromatin structure and affects DNA replication, repair, and activation of gene transcription [124]. *Histone acetyltransferase 1 (HAT1)* partially localizes to the cytoplasm and deacetylates newly synthesized histone H4 on lysines 5 and 12 (H4K5, H4K12) [125]. *HAT1* is involved in several biological processes including chromatin assembly, DNA replication, DNA repair, cell proliferation and glucose metabolism [124].

Liu et al. investigated the relationship between miR-486-5p and ATP-binding cassette transporter A1 (ABCA1)-mediated cholesterol efflux in macrophage-derived foam cells [126]. ABCA1 expression and atherogenesis are regulated by epigenetic modifications [127], but ABCA1 is not a predicted target of miR-486-5p. However, in this study miR-486-5p directly targeted *HAT1* by luciferase reporter assay. Treatment of macrophage-derived foam cells with miR-486-5p mimic decreases *HAT1* expression, decreases H4K5/H4K12 acetylation, and blocks cholesterol efflux. *HAT1* overexpression increases ABCA1 expression, while miR-486-5p mimic inhibits ABCA1 expression [126]. Further supporting the role of miR-486-5p in atherosclerosis, Apoe^−−^/e^−−^ mice treated with exosomes from BM-derived macrophages (enriched in miR-486-5p) develop atherosclerotic lesions with macrophage foam cells [104]. Together, these studies suggest that macrophage miR-486-5p may promote atherosclerosis.

The relationship between miR-486-5p and *HAT1* has also been evaluated in the context of chronic obstructive pulmonary disease (COPD). Zhang et al. reported that miR-486-5p is upregulated in lung tissues of patients with COPD compared to smokers without COPD, and in alveolar macrophages and peripheral monocytes of COPD patients and smokers compared to healthy non-smokers [128]. Rat pulmonary macrophages exposed to cigarette smoking extract upregulate endogenous miR-486-5p and toll-like receptor 4 (TLR4), a known inflammatory trigger, and downregulate *HAT1*, a direct miR-486-5p target. miR-486-5p negatively regulates *HAT1* expression in rat pulmonary macrophages, and *HAT1* suppression increases expression of TLR4 and inflammatory cytokines [128]. miR-486-5p may therefore play a pathological role in COPD by regulating TLR-4 triggered inflammation via its target *HAT1*.

## miR-486-5p targets in development

In skeletal muscle development, the transcription factor *Pax7* is expressed in muscle satellite cells, and is downregulated in activated satellite cells for differentiation [129]. In C2C12 myoblasts, Dey et al.showed that miR-486-5p expression is upregulated during myoblast differentiation [24]. miR-486-5p overexpression accelerates myoblast differentiation, while miR-486-5p inhibition delays differentiation with persistent expression of *Pax7* protein, which is a target of miR-486-5p. Thus, miR-486-5p promotes myogenesis by targeting and downregulating *Pax7*, a transcription factor required for satellite cell biogenesis and survival [24].

In hematopoiesis, miR-486-5p expression is upregulated throughout erythroid differentiation [25, 26]. miR-486-5p overexpression in cord blood CD34+ cells (megakaryocyte–erythroid progenitors) enhances cell growth, erythroid differentiation and cell survival, while miR-486-5p inhibition suppresses these processes [130]. miR-486-5p inhibition also upregulates *PTEN* and *FoxO1* protein expression, decreases p-Akt, and promotes apoptosis and cell growth inhibition. *FoxO1* knockdown rescued the effects of miR-486-5p inhibition but did not influence erythroid differentiation. Thus contributing targets of miR-486-5p other than *FoxO1* remain to be uncovered [130]. The role of miR-486-5p in erythropoiesis has been further characterized in homozygous miR-486-5p knockout mice [25]. The mice are viable, but display defects within the erythroid lineage in bone marrow and spleen, with accumulation of early stage erythroblasts, and a reduced proportion of mature erythrocytes. When subjected to oxidative stress, peripheral blood from miR-486-5p knockout mice shows a greater reduction of red blood cells and increased proportion of reticulocytes [25].

miR-486-5p is also implicated in neurogenesis. Dori et al. isolated discrete populations of neural progenitor cells (proliferative progenitors that expand the stem cell pool and differentiative progenitors that generate neurons) and profiled global miRNA expression during cortical development [131]. miR-486-5p expression is transiently downregulated in differentiative progenitors compared with proliferative progenitors and neurons, termed an ‘off-switch’ transcript. In utero delivery of a locked nucleic acid miR-486-5p inhibitor to mice at embryonic day 13.5 (the developmental stage, where cortical progenitor cells are mostly proliferative progenitors) increases the proportion of cortical progenitor cells at the expense of neurons without affecting neuron migration or survival [131]. These findings suggest that miR-486-5p regulates cell fate in neurogenesis, but the precise targets of miR-486-5p in cortical development remain unknown. Thus, understanding the mechanisms by which miR-486-5p regulates developmental processes could provide additional insight into its role in disease states and identify new targets and pathways for further study.

## Conclusions

**Fig. 4:**
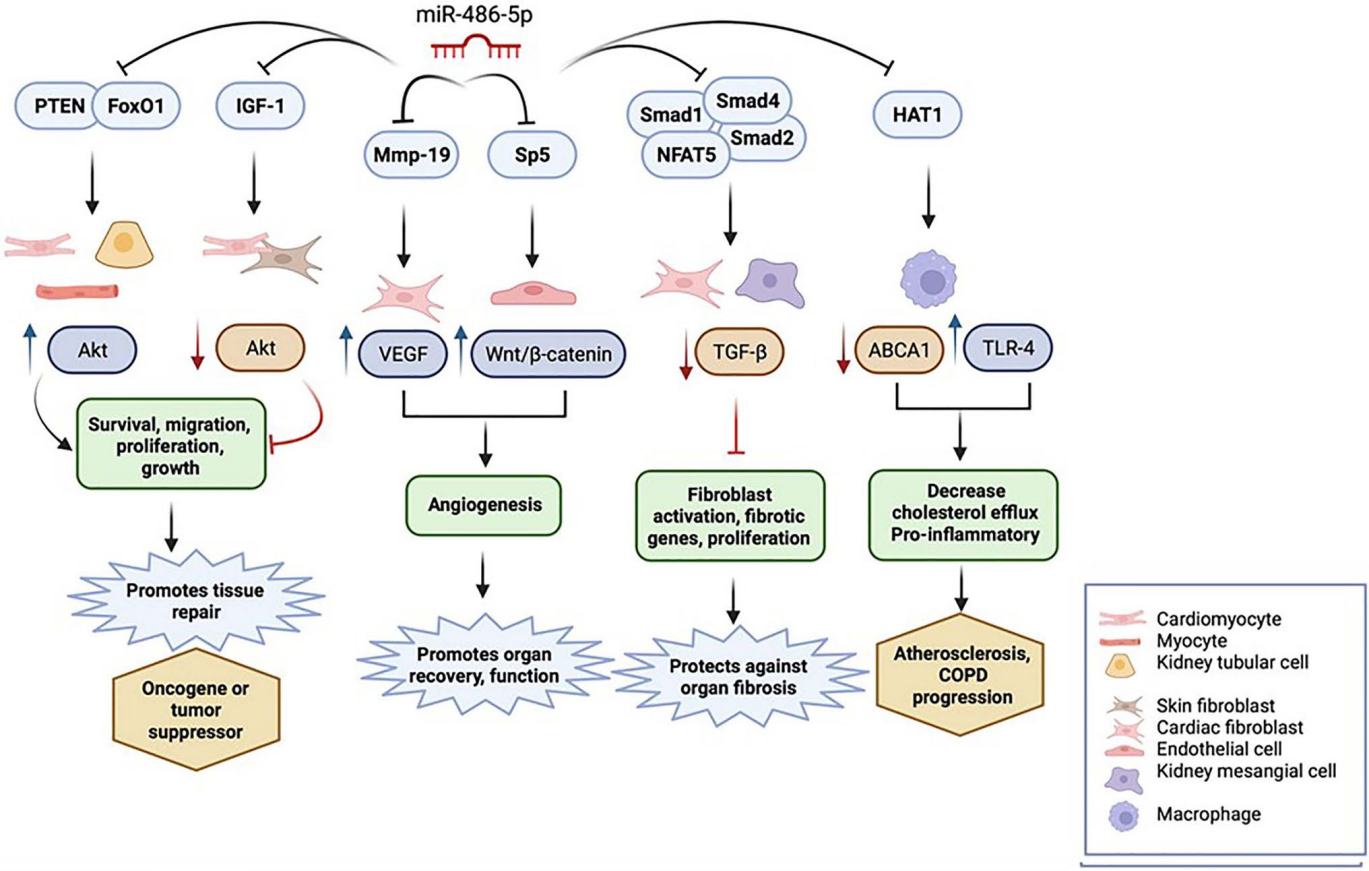
Overview of miR-486-5p target genes, affected signaling pathways, cellular processes, and possible biological effects. miR-486-5p has therapeutic potential in non-malignant diseases by modulating signaling pathways that control critical cellular processes that are involved in tissue regeneration, recovery of organ function, prevention of adverse long-term consequences after organ injury, and organ fibrosis. *ABCA1* ATP binding cassette subfamily A member 1*, COPD* chronic obstructive pulmonary disease, *HAT-1* histone acetyltransferase 1, *IGF-1* insulin-like growth factor-1, *Mmp-19* matrix metalloproteinase-19, *NFAT5* nuclear factor of activated T cells-5, *OA* osteoarthritis, *PI3K* phosphatidylinositol-3-kinase, *PTEN* phosphatase and tensin homolog, *TGF-ß* transforming growth factor-ß, *TLR-4* toll-like receptor-4, *VEGFA* vascular endothelial growth factor-A (created with BioRender.com)

miR-486-5p is implicated in several non-malignant diseases as an important regulator of critical signaling pathways including *PTEN*/Akt, *IGF-1, MMP-19*/VEGF, *Smad*-dependent TGF-β, and Sp5/Wnt/β-catenin, affecting biological processes including apoptosis, cellular migration and proliferation, angiogenesis, and fibrosis (summarized in Fig. 4). miR-486-5p also regulates epigenetic modifications by targeting and downregulating *HAT1*. However, varying biological effects of miR-486-5p have been reported depending on the targeted genes, which may also be specific to cell type and injury model. In addition to its validated 3′UTR targets that are downregulated by post-transcriptional gene silencing via the cytoplasmic RISC pathway, miR-486-5p also localizes to the nucleus, and thus may affect gene expression beyond classical post-transcriptional gene regulation.

Pre-clinical studies support miR-486-5p as a promising therapy for several non-malignant diseases, including cardiac and kidney disorders, due to its proliferative, pro-angiogenic, anti-apoptotic, and anti-fibrotic effects. miR-486-5p protects against cardiac ischemic injury and targets *PTEN,* suppressing cardiomyocyte apoptosis in cardiac IRI [17, 81], improves cardiac function and promotes angiogenesis after myocardial infarction associated with targeting *Mmp-19* in cardiac fibroblasts [65], and reduces IgE-mediated cardiac fibrosis by targeting *Smad1* [19]. miR-486-5p also protects against ischemic kidney injury and targets *PTEN* [16, 45], associated with decreased expression of select genes involved in apoptosis and the TNF inflammatory pathway, and may confer a protective anti-fibrotic role in diabetic kidney disease by targeting *NFAT5* [21]. Although there are no human clinical trials involving miR-486-5p therapy to date, clinical trials are underway for other miRNAs [132]. For instance, a phase I trial of intravenous liposomal miR-34a mimic administered to patients with advanced solid tumors was found to target tumors but unfortunately led to serious immune-mediated adverse events, resulting in early trial termination [133]. In Alport nephropathy—a hereditary nephropathy—Phase 1 trials involving use of the miR-21 inhibitor RG-012 have been completed and will move to Phase 2 [132]. Accordingly, miR-486-5p has potential as a bio-therapeutic agent in several non-malignant diseases. Nonetheless, studies addressing the safety profile and longer term effects of miR-486-5p delivery are warranted, since miR-486-5p affects cell cycle kinetics [14], its expression is tightly regulated in growth and development [11], and dysregulated expression has been associated with malignancy potential [9].

## Supplementary Information

Below is the link to the electronic supplementary material.

Supplementary file1 (DOCX 34 KB)

## Data Availability

Not applicable.
