# Supplementary material for: miRNA-486-5p: signaling targets and role in non-malignant disease
Source: Cell Mol Life Sci. 2022 Jun 22;79(7):376. doi: 10.1007/s00018-022-04406-y (PMC9217846; doi:10.1007/s00018-022-04406-y)
Supplement: Supplementary file 1 — Supplementary file1 (DOCX 34 KB) [file 18_2022_4406_MOESM1_ESM.docx]

\

Table 1. Differential expression of miR-486-5p in human non-malignant diseases

| **Reference** | **Study population** | **Summary of results** |
| --- | --- | --- |
| **Cardiac diseases** | | |
| Zhao H *et al* (2021) | Chronic heart failure (N=57) vs healthy controls (N=87) | Serum miR-486-5p (i) decreased in heart failure patients (ii) negatively correlated with NT-proBNP |
| Berkan Ö *et al* (2019) | Diseased coronary atherosclerotic plaques vs healthy arteries from patients with CAD (N=14) | miR-486-5p (i) downregulated in diseased coronary atherosclerotic plaques; (ii) negatively correlated with triglyceride levels |
| Mukai N *et al* (2018) | Erythrocytes from children with cyanotic and acyanotic heart disease vs healthy controls (N=10 per group) | -Erythrocyte miR-486-5p increased in cyanotic and acyanotic heart disease vs healthy controls |
| **Kidney diseases and kidney transplantation** | | |
| Regmi A *et al* (2019) | Patients with uncomplicated T2DM (N=50), T2DM with DKD (N=42), vs healthy (N=25) | Serum miR-486-5p (i) downregulated in T2DM+DKD vs healthy controls; (ii) negatively correlated with albuminuria, blood pressure, lipids, and glucose/glycated Hgb |
| Argyropoulos C *et al* (2015) | Patients with T1DM without microalbuminuria who: did not develop nephropathy (N=10) vs developed microalbuminuria (N=17) | Urinary miR-486-5p part of miRNA signature that predicted development of microalbuminuria in T1DM |
| Duan Z-Y *et al* (2016) | Patients with IgAN (N=93), other glomerular disease (N=40), and healthy controls (N=82) | -Urinary sediment and microvesicle-associated miR-486-5p increased in IgAN  -No difference in blood erythrocyte miR-486-5p between IgAN and controls |
| Baker MA *et al* (2017) | Patients with (i) DN (ii) FSGS (iii) IgAN (iv) MPGN (v) control group (N=19-23/disease, N=14 controls) | -Glomerular miR-486-5p decreased in DN, MPGN, IgAN  -Proximal tubular miR-486-5p decreased in DN, IgAN, FSGS |
| Iwasaki K *et al* (2017) | Kidney transplant patients: (1) DSA (-) no CAMR (N=22);  (2) DSA (+) no CAMR (N=13); (3) DSA (+) subclinical CAMR (N=10); (4) DSA (+) clinical CAMR (N=9) | -PBMC and whole blood miR-486-5p: upregulated after DSA production  -whole blood miR-486-5p: upregulated in early (subclinical) CAMR |
| Gómez-Dos-Santos V *et al* (2019) | DCD/ECD kidney transplants selected according to DGF development and graft function at 1 year | Hypothermic machine perfusion fluid miR-486-5p associated with DGF |
| **Lung diseases** | | |
| Zhang J *et al* (2020) | BAL fluid from patients with COPD (N=36) and controls (N=14)  -Patient blood: non-smokers (N=33), smokers (N=42), COPD (N=53);  -human miRNA datasets (i) lung tissue from COPD (N=19) vs smokers without COPD (N=8) (ii) small airway epithelium from non-smokers (N=9) vs smokers (N=10) | miR-486-5p increased in (i) lung tissue from COPD patients (ii) small airway epithelium of smokers (iii) alveolar macrophages of COPD patients (iv) peripheral monocytes of COPD patients and smokers without COPD |
| Ideozu JE *et al* (2019) | Patients with cystic fibrosis vs heathy controls (N=10 per group) | Plasma extracellular miR-486-5p upregulated in patients with cystic fibrosis |
| **Infectious diseases** | | |
| Sun B *et al* (2021) | Critically ill patients with sepsis (N=108), pneumonia without sepsis (N=60), and controls (N=101) | Serum miR-486-5p (i) upregulated in sepsis vs pneumonia and controls (ii) positively associated with disease severity, inflammatory markers, and survival |
| De Gonzalo-Calvo *et al* (2021) | Patients hospitalized with COVID-19: ward (N=43) vs ICU (N=36); ICU survivors (N=20) vs non-survivors (N=16) | Circulating miR-486-5p (i) decreased in ICU patients vs ward and (ii) not associated with ICU survival |
| **Endocrine diseases and fertility** | | |
| Zaki MB *et al* (2019) | Adult male patients with metabolic syndrome (N=55) vs healthy controls (N=20) | Serum miR-486-5p upregulated in male patients with metabolic syndrome and positively correlated with waist circumference |
| Prats-Puig A *et al* (2013) | Prepubertal caucasian children, obese (N=40) and lean (N=85) | Plasma miR-486-5p increased in obese children and associated with obesity measures |
| Karere GM *et al* (2021) | Adolescents with obesity (N=68; 60% male) | Whole blood miR-486-5p upregulated in females and downregulated in males |
| Flórez CAR *et al* (2019) | Systematic review of 7 studies (N=361 obese/overweight children, N=323 children with normal weight) | miR-486-5p reported in 2/7 studies, and upregulated in obese children |
| Matsha TE *et al* (2018) | Female patients with T2DM, impaired glucose tolerance, vs normal glucose tolerance (N=12 per group) | Whole blood miR-486-5p downregulated in T2DM vs normal glucose tolerance, and in T2DM vs impaired glucose tolerance |
| Flowers E *et al* (2015) | Patients without DM, either insulin resistant (N=75) or insulin sensitive (N=18) | Plasma miR-486-5p increased in patients with insulin resistance and positively correlated with response to TZD treatment |
| Bouchareychas L *et al* (2021) | Patients with PAD (N=5) vs DM without vascular disease (N=9) vs DM+PAD (N=5) vs healthy controls (N=7) | -miR-486-5p upregulated in circulating exosomes from patients with DM+PAD vs DM and healthy controls |
| Zhou Y *et al* (2018) | Patients with (i) subclinical hypothyroidism + SA (N=21) (ii) subclinical hypothyroidism (N=24) (iii) SA (N=19) (iv) controls (N=18) | Serum miR-486-5p (i) increased in patients with subclinical hypothyroidism + SA vs SA or controls (ii) no difference between subclinical hypothyroidism and controls |
| Butler AE *et al* (2020) | Patients with anovulatory PCOS (N=29) vs controls (N=24) | Plasma miR-486-5p upregulated in PCOS and not associated with BMI, androgen levels, or insulin resistance |
| Shi L *et al* (2015) | Patients with PCOS (N=24) vs non-PCOS women (N=24) | miR-486-5p from oocyte cumulus cells downregulated in PCOS patients |
| **Cerebrovascular and neurological diseases** | | |
| Prabhakar P *et al* (2017) | Patients with vascular dementia (N=204) vs controls (N=200) | Plasma miR-486-5p upregulated with small vessel vascular dementia |
| Lopes KDP *et al* (2018) | Patients with cerebral aneurysm post-hemorrhage with and without vasospasm (N=14, 13) vs controls (N=6) | Circulating miR-486-5p (i) downregulated in aneurysmal SAH vs controls |
| Kurz A *et al* (2021) | Colonic biopsies from patients with PD (N=13) vs controls (N=17) | miR-486-5p (i) upregulated in colonic biopsies from patients with PD vs controls (ii) correlated with age and disease severity |
| Hoss AG *et al* (2015) | Patients with symptomatic HD (N=26), asymptomatic HD gene carriers (N=4), controls (N=8) | Plasma miR-486-5p upregulated in symptomatic HD patients |
| Ghahramani Seno MM *et al* (2011) | Lymphoblastoid cells from patients (N=20) confirmed diagnosis of autism spectrum disorder (ASD), 22 controls (unaffected siblings) | miR-486-5p from lymphoblastoid cells from patients with ASD is differentially regulated, but the direction of change is inconsistent |
| **Arthritic diseases** | | |
| Kong R *et al* (2017) | Patients with knee OA (N=100) and healthy controls (N=100) | Plasma miR-486-5p (i) upregulated in knee OA (ii) independent factor for knee OA risk (iii) associated with disease severity |
| **Primary muscle diseases** | | |
| Eisenberg I *et al* (2007) | Skeletal muscle specimens from 10 groups of primary muscle disorders and human skeletal muscle controls | Skeletal muscle miR-486-5p downregulated only in DMD |

Abbreviations: BAL – bronchoalveolar lavage; CAD – coronary artery disease; CAMR – chronic antibody-mediated rejection; COPD – chronic obstructive pulmonary disease; DCD/ECD – donation after circulatory death, expanded criteria donor; DGF – delayed graft function; FSGS – focal segmental glomerulosclerosis; DKD – diabetic kidney disease ; DMD – Duchenne muscular dystrophy; DN – diabetic nephropathy; DSA – donor specific antibody; HD – Huntington’s disease; ICU – intensive care unit; IgAN – IgA nephropathy; MPGN – membranoproliferative glomerulonephritis; NT-proBNP – N-terminal pro-hormone B-type natriuretic peptide; OA – osteoarthritis; PAD – peripheral arterial disease; PBMC – peripheral blood mononuclear cell; PCOS: polycystic ovary syndrome; PD – Parkinson’s disease; SA – spontaneous abortion; SAH – subarachnoid; T1DM – type 1 diabetes; T2DM – type 2 diabetes; TZD – thiazolidinedione

References

1. Zhao H, Yang H, Geng C, *et al* (2021) Elevated IgE promotes cardiac fibrosis by suppressing miR-486-5p; *Theranostics*; **11**:7600-7015. <https://doi.org/10.7150/thno.47845>
2. Berkan Ö, Arslan S, Lalem T, *et al* (2019) Regulation of microRNAs in coronary atherosclerotic plaque; *Epigenomics;* **11**(12):1387-139. <https://doi.org/10.2217/epi-2019-0036>
3. Mukai N, Nakayama Y, Murakami S, *et al* (2018) Potential contribution of erythrocyte microRNA to secondary erythrocytosis and thrombocytopenia in congenital heart disease. *Pediatric Research*; **83**(4):866-873. <https://doi.org/10.1038/pr.2017.327>
4. Regmi A, Liu G, Zhong X, *et al* (2019) Evaluation of Serum microRNAs in Patients with Diabetic Kidney Disease: A Nested Case-Controlled Study and Bioinformatics Analysis. *Med Sci Monit*; **25**:1699-1708. <https://doi.org/10.12659/MSM.913265>
5. Argyropoulos C, Wang K, Bernardo J, *et al* (2015) Urinary MicroRNA Profiling Predicts the Development of Microalbuminuria in Patients with Type 1 Diabetes.  *J Clin Med*; **4**:1498-1517. <https://doi.org/10.3390/jcm4071498>
6. Duan Z-Y, Cai G, Bu R, *et al* (2016) Selection of urinary sediment miRNAs as specific biomarkers of IgA nephropathy. *Scientific Reports*; **6**:23498. <https://doi.org/10.1038/srep23498>
7. Baker MA, Davis SJ, Liu P, *et al* (2017) Tissue-Specific MicroRNA Expression Patterns in Four Types of Kidney Disease. *J Am Soc Nephrol*; **28**:2985-2992. <https://doi.org/10.1681/ASN.2016121280>
8. Iwasaki K, Yamamoto T, Inanaga Y, *et al* (2017) MiR-142-5p and miR-486-5p as biomarkers for early detection of chronic antibody-mediated rejection in kidney transplantation. *Biomarkers*; **22**(1):45-54. <https://doi.org/10.1080/1354750X.2016.1204000>
9. Gómez-Dos-Santos V, Ramos-Muñoz E, García-Bermejo ML, *et al* (2019) MicroRNAs in Kidney Machine Perfusion Fluid as Novel Biomarkers for Graft Function. Normalization Methods for miRNAs Profile Analysis. *Transplantation Proceedings*; **51**:307-310. <https://doi.org/10.1016/j.transproceed.2018.09.019>
10. Zhang J, Xu Z, Kong L, *et al* (2020) miRNA-486-5p Promotes COPD Progression by Targeting HAT1 to Regulate the TLR4-Triggered Inflammatory Response of Alveolar Macrophages. *International Journal of Chronic Obstructive Pulmonary Disease*; **15**:2991-3001. <https://doi.org/10.2147/COPD.S280614>
11. Ideozu JE, Zhang X, Rangaraj V, *et al* (2019) Microarray profiling identifies extracellular circulating miRNAs dysregulated in cystic fibrosis. *Scientific Reports*; **9**:15483. <https://doi.org/10.1038/s41598-019-51890-7>
12. Sun B and Guo S (2021) miR-486-5p Serves as a Diagnostic Biomarker for Sepsis and Its Predictive Value for Clinical Outcomes. *Journal of Inflammation Research*; **14**:3687-3695. <https://doi.org/10.2147/JIR.S323433>
13. De Gonzalo-Calvo D, Benítez ID, Pinilla L, *et al* (2021) Circulating microRNA profiles predict the severity of COVID-19 in hospitalized patients. *Translational Research*; **236**:147-159. <https://doi.org/10.1016/j.trsl.2021.05.004>
14. Zaki MB, Abulsoud AI, Elsisi AM, *et al* (2019) Potential role of circulating microRNAs (486-5p, 497, 509-5p and 605) in metabolic syndrome Egyptian male patients. *Diabetes, Metabolic Syndrome and Obesity: Targets and Therapy*; **12**:601-611. <https://doi.org/10.2147/DMSO.S187422>
15. Prats-Puig A, Ortega FJ, Mercader JM, *et al* (2013) Changes in Circulating MicroRNAs Are Associated With Childhood Obesity. *J Clin Endocrinol Metab*; **98**(10):E1655-E1660. <https://doi.org/10.1210/jc.2013-1496>
16. Karere GM, Cox LA, Bishop AC, *et al* (2021) Sex Differences in MicroRNA Expression and Cardiometabolic Risk Factors in Hispanic Adolescents with Obesity. *J Pediatr*; **235:**138-143. <https://doi.org/10.1016/j.jpeds.2021.03.070>
17. Matsha TE, Kengne AP, Hector S, *et al* (2018) MicroRNA profiling and their pathways in South African individuals with prediabetes and newly diagnosed type 2 diabetes mellitus. *Oncotarget*; **9**(55):30485-30498. <https://doi.org/10.18632/oncotarget.25271>
18. Flowers E, Aouizerat BE, Abbasi F, *et al* (2015) Circulating MicroRNA-320a and MicroRNA-486 Predict Thiazolidinedione Response: Moving Towards Precision Health for Diabetes Prevention. *Metabolism*; **64**(9):1051-1059. <https://doi.org/10.1016/j.metabol.2015.05.013>
19. Bouchareychas L, Duong P, Phu TA, *et al* (2021) High glucose macrophage exosomes enhance atherosclerosis by driving cellular proliferation & hematopoiesis. *iScience*; **24**:102847. <https://doi.org/10.1016/j.isci.2021.102847>
20. Zhou Y, Wang X, Zhang Y, *et al* (2018) Circulating MicroRNA Profile as a Potential Predictive Biomarker for Early Diagnosis of Spontaneous Abortion in Patients with Subclinical Hypothyroidism. *Front Endocrinol*; **9**:128. <https://doi.org/10.3389/fendo.2018.00128>
21. Butler AE, Ramachandran V, Sathyapalan T, *et al.* (2020) microRNA Expression in Women With and Without Polycystic Ovarian Syndrome Matched for Body Mass Index. *Front Endocrinol*; **11**:206. <https://doi.org/10.3389/fendo.2020.00206>
22. Shi L, Liu S, Zhao W, *et al* (2015) miR-483-5p and miR-486-5p are down-regulated in cumulus cells of metaphase II oocytes from women with polycystic ovary syndrome. *Reprod Biomed Online*; **31**:565-572. <https://doi.org/10.1016/j.rbmo.2015.06.023>
23. Prabhakar P, Chandra SR, and Christopher R (2017) Circulating microRNAs as potential biomarkers for the identification of vascular dementia due to cerebral small vessel disease*. Age and Ageing*; **46**:861-864*.* <https://doi.org/10.1093/ageing/afx090>
24. Lopes KDP, Vinasco-Sandoval T, Vialle RA, *et al* (2018) Global miRNA expression profile reveals novel molecular players in aneurysmal subarachnoid haemorrhage. *Scientific Reports*; **8**:8786. <https://doi.org/10.1038/s41598-018-27078-w>
25. Kurz A, Kumar R, Northoff BH, *et al* (2021) Differential expression of gut miRNAs in idiopathic Parkinson’s disease. *Parkinsonism and Related Disorders*; **88**:46-50. <https://doi.org/10.1016/j.parkreldis.2021.05.022>
26. Hoss AG, Lagomarsino VN, Frank S, *et al* (2015) Study of Plasma-Derived miRNAs Mimic Differences in Huntington’s Disease Brain. *Mov Disord*; **30**(14):1961-1964. <https://doi.org/10.1002/mds.26457>
27. Ghahramani Seno MM, Hu P, Gwadry FG, *et al* (2011) Gene and miRNA expression profiles in autism spectrum disorders. *Brain Res.* **1380**:85-97

<https://doi.org/10.1016/j.brainres.2010.09.046>

1. Kong R, Gao J, Si Y, *et al* (2017) Combination of circulating miR-19b-3p, miR-122-5-p and miR-486-5p expressions correlates with risk and disease severity of knee osteoarthritis. *Am J Transl Res*; **9**(6):2852-2864
2. Eisenberg I, Eran A, Nishino I, *et al* (2007) Distinctive patterns of microRNA expression in primary muscle disorders. *PNAS*; **104**(43):17016-17021. <https://doi.org/10.1073/pnas.0708115104>
